# Association Between Triglyceride Glucose Index and Risk of New-Onset Diabetes Among Chinese Adults: Findings From the China Health and Retirement Longitudinal Study

**DOI:** 10.3389/fcvm.2020.610322

**Published:** 2020-11-27

**Authors:** Chao-lei Chen, Lin Liu, Kenneth Lo, Jia-yi Huang, Yu-ling Yu, Yu-qing Huang, Ying-qing Feng

**Affiliations:** ^1^Department of Cardiology, Guangdong Cardiovascular Institute, Guangdong Provincial Key Laboratory of Coronary Heart Disease Prevention, Guangdong Provincial People's Hospital, Guangdong Academy of Medical Sciences, Guangzhou, China; ^2^Department of Epidemiology, Centre for Global Cardiometabolic Health, Brown University, Providence, RI, United States; ^3^Department of Applied Biology and Chemical Technology, The Hong Kong Polytechnic University, Hung Hom, China

**Keywords:** diabetes mellitus, association, CHARLS, predict, triglyceride glucose index

## Abstract

The triglyceride glucose (TyG) index has been proposed to be a surrogate of insulin resistance. In the present study, we aimed to examine the relationship between TyG index and the risk of incident diabetes in middle-age and older adults in China using nationally representative data from the China Health and Retirement Longitudinal Study 2011–2015. Information on socio-demographics, medical background, anthropometric measurement, and laboratory information were collected. The association between TyG index and diabetes was examined by Cox proportional hazards models and restricted cubic spline regression, and the results were presented in hazard ratio (HR) with 95% confidence interval (CI). Subgroup analyses were also conducted to examine potential interactions between demographics and TyG index. Among 7,428 participants, 791 (10.6%) of them developed diabetes over 3.4 years of follow-up. The multivariate HR for every SD increment in TyG index was 1.22 (95% CI, 1.14–1.31). When comparing to the lowest quartile of TyG index, the multivariate HRs for new-onset diabetes were 1.22 (0.96–1.54) for Q2, 1.61 (1.28–2.01) for Q3, and 1.73 (1.38–2.16) for Q4 (*P* for trend <0.001). The restricted cubic spline regression also showed a linear association. No interaction was found between subgroup variables and the association between TyG index and the risk of diabetes. In conclusion, higher TyG index associated with the elevated risk of new-onset diabetes in middle-aged and older adults.

## Introduction

Diabetes is associated with an increased risk of cardiovascular disease and all-cause mortality, imposing a huge burden to public health (1). In the past few decades, the number of adults with diabetes has considerably increased, especially in low and middle-income countries (2). According to the International Diabetes Federation, China has the largest number of diabetic patients, reaching 114.4 million in 2017 (3). Therefore, identifying individuals at high risk for developing diabetes is of major importance to reduce the incidence rate and related complications.

Insulin resistance (IR) is a major pathophysiological pathway of type 2 diabetes development and may appear about two decades prior to the formal diagnosis (4, 5). A recent study indicated that IR was more closely associated with the risk of incident diabetes among Chinese adults than β-cell dysfunction, which is another pivotal pathological feature of diabetes (6). Additionally, in the past four decades, China has experienced disruptive transitions of dietary patterns and has witnessed a massive rise in with the rate of obesity, which is major factor for the progression of IR (7, 8). Moreover, the assessment of IR status is essential to identify individuals with high risk of diabetes. The traditional approach to measure IR, such as the homeostasis model assessment of IR (HOMA-IR), is time-consuming and costly for daily practice and in large epidemiological studies.

Recently, the triglyceride glucose (TyG) index, the product of triglyceride (TG) and fasting blood glucose (FBG), has attracted increasing attention as a simple indicator of IR owing to its good correlation with HOMA-IR and better performance to examine insulin sensitivity (9). Several studies have examined the association of TyG index and diabetes in Asia and western populations (10–15). However, the results were inconclusive and limited due to either the cross-sectional design (14), had small sample size (12) or being performed among selected population (11, 15). Therefore, we designed a prospective cohort study using nationally representative data from the China Health and Retirement Longitudinal Study (CHARLS) to explore the relationship between TyG index and the risk of new-onset diabetes.

## Methods

### Study Design and Participants

**Figure 1:**
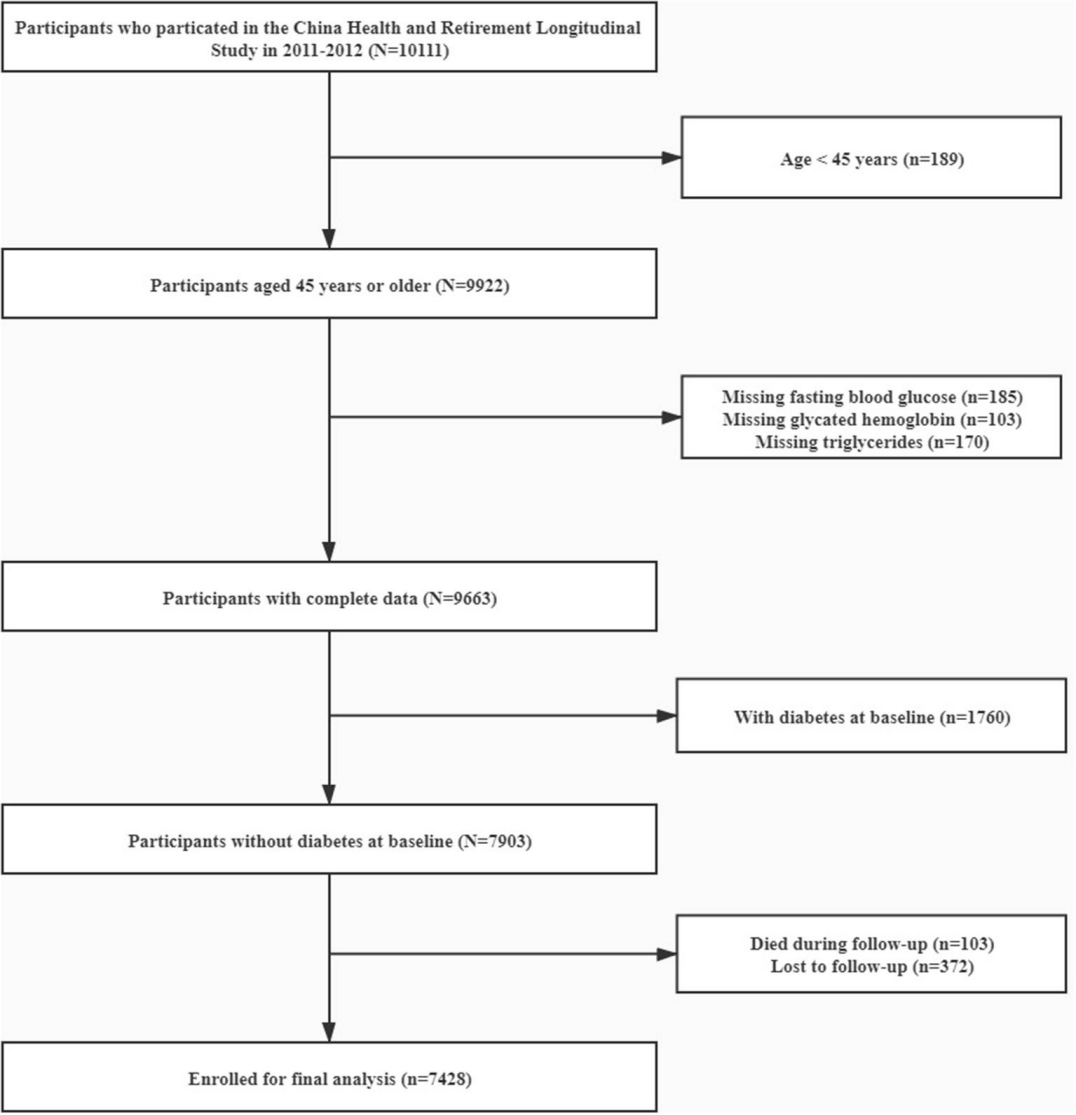
Flow chart of study participants.

CHARLS is an ongoing longitudinal survey to examine the social, economic and health status of community residents aged 45 years or older in China. Details of the study design of CHARLS have been described elsewhere (16). Briefly, the CHARLS adopted a multistage probability sampling and investigated 17,708 individuals in 28 provinces through random selection of 10,257 households to cover the overall population in China in the first wave (W1, 2011–2012). The response rate by provinces was up to 81% in the baseline survey. Information on socio-demographics, physical and biological assessments, and health-related information of participants were collected via standardized interviews. To date, the follow-up surveys have been conducted twice, including the second wave (W2) in 2013 and the third wave (W3) in 2015. For the present study, 10,111 individuals have been enrolled with data in laboratory measurement at W1. Participants aged <45, with missing data on TG, FBG, and glycated hemoglobin were excluded. After further excluding 1,760 participants with diabetes at baseline we also excluded subjects who died (*n* = 103) or lost follow-up (*n* = 372) in the subsequent waves (W2 and W3) of the study. Finally, 7,428 participants were enrolled for the final analysis (Figure 1). The Ethics Review Committee of Peking University approved CHARLS (IRB00001052–11015) and all participants have provided informed consent before participation.

### Data Collection and Definitions

Trained researchers interviewed participants in their homes using computer-assisted technology to collect sociodemographic information [including age, gender, education level (primary school or lower, secondary school, and higher), and marriage status (current married or not)], health behavior (including current habits of smoking and alcohol drinking), medical history [including self-reported hypertension, diabetes, and cardiovascular disease (CVD)] and medication usage (including antihypertensive drugs, antidiabetic drugs, and lipid-lowering drugs).

Anthropometric indicators included systolic blood pressure (SBP), diastolic blood pressure (DBP), body mass index (BMI), and waist circumference (WC). Blood pressure were measured three times in a seated position by trained nurses using the HEM-7200 electronic monitor (Omron, Dalian, Japan). Hypertension was defined as SBP≥140 mmHg or DBP ≥90 mmHg or self-reported prior diagnosis of hypertension by a doctor or using antihypertensive drugs in the past 2 weeks (17). Height and WC measurement were accurate to 0.1 cm and 0.1 kg, respectively.

These samples were transported from all study sites to Beijing and were stored at −80°C at the Chinese Center for Disease Control and Prevention. The determination of FBG, Hemoglobin A1c, TG, total cholesterol (TC), low-density lipoprotein cholesterol (LDL-C), and high-density lipoprotein cholesterol (HDL-C) were conducted by trained research staff. TyG index was calculated as ln [TG (mg/dl) × FBG (mg/dl)/2] (18). At baseline and follow-up, diabetes was defined as FBG > 125 mg/dL or Hemoglobin A1c >6.5%, self-reported prior diagnosis of diabetes by a doctor or using antidiabetic medications. Participants whose FBG was at 100–125 mg/dL or Hemoglobin A1c was at 5.7–6.4% were classified as having prediabetes (19). Participants without diabetes or prediabetes were defined as normoglycemia.

### Statistical Analysis

Data was presented as mean and standard deviation (SD) for continuous variables, and percentage for categorical variables. All continuous variables have followed a Gaussian distribution according to Kolmogorov-Smirnov test (*P* > 0.1). Baseline characteristics and the rate of diabetes development were grouped by quartiles of TyG index (Q1, Q2, Q3, Q4) and compared using the One-Way ANOVA, Kruskal-Wallis H test or chi-square tests, as appropriate. We initially built Cox proportional hazards models to estimate HR with 95% confidence interval (CI) of diabetes for TyG index as continuous (per SD increment) or categorical (quartiles) variables. Three Cox regression models were fitted. Model 1 only included TyG index. Model 2 was adjusted for age and gender. Fully adjusted model (Model 3) was adjusted for age, gender, education, marriage, smoking, alcohol drinking, BMI, WC, SBP, history of hypertension, history of CVD, and the usage of lipid-lowering drugs. Next, the shape of association between TyG index and incident diabetes was examined by multivariate restricted cubic spline model. We chose three knots at quartiles 25th, 50th, and 75th. Finally, we performed subgroup analyses of Cox proportional hazards models including age (<65 or ≥65 years), gender (male or female), BMI (<25 or ≥25 kg/m^2^), glycemic status (normoglycemia or prediabetes at baseline), FBG (<100 or ≥100 mg/dL), and the level of TG (<100 or ≥100 mg/dL). *P* < 0.05 was considered statistically significant. R version 3.3.2 (R Foundation for Statistical Computing, Vienna, Austria) was used for all statistical analyses.

## Results

### Baseline Characteristics of Study Participants

The baseline characteristics of all participants according to quartiles of TyG index and the proportion of diabetes development were summarized in Tables 1, 2, respectively. The present study included 7,428 participants (male: 46.5% and mean age: 59.3 years). The mean value TyG index was 8.56. Among quartiles of TyG index, we observed significant differences in all baseline covariates except for marriage status and education level (Table 1). When compared with participants without diabetes during follow-up, subjects who developed diabetes were older in age, having lower education level, less likely to be married, having higher levels of SBP, DBP, BMI, WC, TG, TC, LDL-C, HDL-C, FBG, Hemoglobin A1c, and TyG index, more likely to have hypertension and CVD, and more likely to use lipid-lowing drugs (Table 2).

**Table 1:** Baseline characteristics of study participants according to quartiles of triglyceride glucose index.

	**Q1**	**Q2**	**Q3**	**Q4**	***P*-value**
Number	1,857	1,857	1,857	1,857	
Age, years	59.56 ± 9.83	59.36 ± 9.36	59.60 ± 9.12	58.67 ± 9.11	0.008
Gender					<0.001
Male	1,007 (54.2)	921 (49.6)	782 (42.1)	747 (40.2)	
Female	850 (45.8)	936 (50.4)	1,075 (57.9)	1,110 (59.8)	
Education					0.338
Primary school or lower	1,325 (71.4)	1,307 (70.4)	1,332 (71.7)	1,281 (69.0)	
Secondary school	358 (19.3)	362 (19.5)	342 (18.4)	400 (21.5)	
Higher	173 (9.3)	187 (10.1)	183 (9.9)	176 (9.5)	
Current married	1,639 (88.3)	1,644 (88.5)	1,632 (87.9)	1,649 (88.8)	0.843
Current smoking	808 (43.5)	753 (40.5)	681 (36.7)	663 (35.7)	<0.001
Current drinking	548 (29.5)	498 (26.8)	403 (21.7)	441 (23.7)	<0.001
SBP, mmHg	126.70 ± 21.07	128.29 ± 20.75	131.30 ± 22.03	133.00 ± 21.63	<0.001
DBP, mmHg	73.69 ± 12.10	74.59 ± 11.96	76.34 ± 12.40	77.70 ± 12.52	<0.001
BMI, kg/m^2^	22.01 ± 3.18	22.83 ± 3.73	23.61 ± 3.67	24.78 ± 3.67	<0.001
WC, cm	80.06 ± 10.74	82.55 ± 11.74	84.59 ± 12.75	87.71 ± 12.49	<0.001
TC, mg/dL	178.15 ± 33.52	188.61 ± 33.88	196.85 ± 36.74	205.84 ± 39.63	<0.001
TG, mg/dL	59.72 ± 13.51	86.81 ± 13.01	121.10 ± 18.03	217.93 ± 88.01	<0.001
LDL-C, mg/dL	107.93 ± 29.13	117.54 ± 30.86	124.06 ± 33.99	118.19 ± 39.17	<0.001
HDL-C, mg/dL	60.47 ± 14.97	55.65 ± 13.98	50.28 ± 12.89	42.27 ± 11.38	<0.001
FBG, mg/dL	93.57 ± 13.31	99.16 ± 10.40	101.03 ± 10.40	106.14 ± 9.98	<0.001
Hemoglobin A1c, %	5.05 ± 0.38	5.08 ± 0.39	5.11 ± 0.41	5.16 ± 0.40	<0.001
TyG index	7.90 ± 0.25	8.35 ± 0.10	8.70 ± 0.11	9.29 ± 0.33	<0.001
Hypertension	568 (30.8)	652 (35.5)	784 (42.7)	888 (48.2)	<0.001
Cardiovascular disease	197 (10.6)	233 (12.6)	249 (13.5)	265 (14.3)	0.006
Lipid-lowering drugs	34 (1.8)	52 (2.8)	76 (4.1)	107 (5.8)	<0.001
New-onset diabetes	123 (6.6)	166 (8.9)	232 (12.5)	270 (14.5)	<0.001

*Data are presented as mean ± standard deviation or number (%)*.

*Q, quartiles; SBP, systolic blood pressure; DBP, diastolic blood pressure; BMI, body mass index; WC, waist circumference; TC, total cholesterol; TG, triglyceride; LDL-C, low-density lipoprotein cholesterol; HDL-C, high-density lipoprotein cholesterol; FBG, fasting blood glucose; TyG, triglyceride glucose*.

**Table 2:** Comparison of baseline characteristics of study participants who developed diabetes or not.

	**Overall**	**Not developed DM**	**Developed DM**	***P*-value**
Number	7,428	6,637	791	
Age, years	59.30 ± 9.36	59.14 ± 9.37	60.68 ± 9.20	<0.001
Gender				0.210
Male	3,457 (46.5)	3,106 (46.8)	351 (44.4)	
Female	3,971 (53.5)	3,531 (53.2)	440 (55.6)	
Education				0.018
Primary school or lower	5,245 (70.6)	4,656 (70.2)	589 (74.6)	
Secondary school	1,462 (19.7)	1,319 (19.9)	143 (18.1)	
Higher	719 (9.7)	661 (10.0)	58 (7.3)	
Current married	6,564 (88.4)	5,888 (88.7)	676 (85.5)	0.008
Current smoking	2,905 (39.1)	2,595 (39.1)	310 (39.2)	0.993
Current drinking	1,890 (25.4)	1,709 (25.7)	181 (22.9)	0.088
SBP, mmHg	129.82 ± 21.51	129.29 ± 21.42	134.25 ± 21.78	<0.001
DBP, mmHg	75.58 ± 12.34	75.29 ± 12.24	77.98 ± 12.92	<0.001
BMI, kg/m^2^	23.31 ± 3.71	23.16 ± 3.62	24.52 ± 4.23	<0.001
WC, cm	83.72 ± 12.28	83.31 ± 12.13	87.18 ± 12.94	<0.001
TC, mg/dL	192.36 ± 37.44	191.85 ± 37.38	196.62 ± 37.70	0.001
TG, mg/dL	121.39 ± 75.40	119.39 ± 73.93	138.21 ± 84.99	<0.001
LDL-C, mg/dL	116.93 ± 34.00	116.63 ± 33.98	119.46 ± 34.07	0.027
HDL-C, mg/dL	52.17 ± 14.98	52.46 ± 14.87	49.74 ± 15.68	<0.001
FBG, mg/dL	99.98 ± 11.98	99.54 ± 11.84	103.65 ± 12.47	<0.001
Hemoglobin A1c, %	5.10 ± 0.40	5.08 ± 0.39	5.25 ± 0.44	<0.001
TyG index	8.56 ± 0.56	8.54 ± 0.55	8.72 ± 0.56	<0.001
Hypertension	2,892 (39.3)	2,483 (37.7)	409 (52.1)	<0.001
Cardiovascular disease	944 (12.8)	807 (12.2)	137 (17.4)	<0.001
Lipid-lowering drugs	269 (3.6)	222 (3.3)	47 (5.9)	<0.001

*Data are presented as mean ± SD or n (%)*.

*Q, quartiles; SBP, systolic blood pressure; DBP, diastolic blood pressure; BMI, body mass index; WC, waist circumference; TC, total cholesterol; TG, triglyceride; LDL-C, low-density lipoprotein cholesterol; HDL-C, high-density lipoprotein cholesterol; FBG, fasting blood glucose; TyG, triglyceride glucose*.

### Hazard Ratios for Incident Diabetes

**Figure 2:**
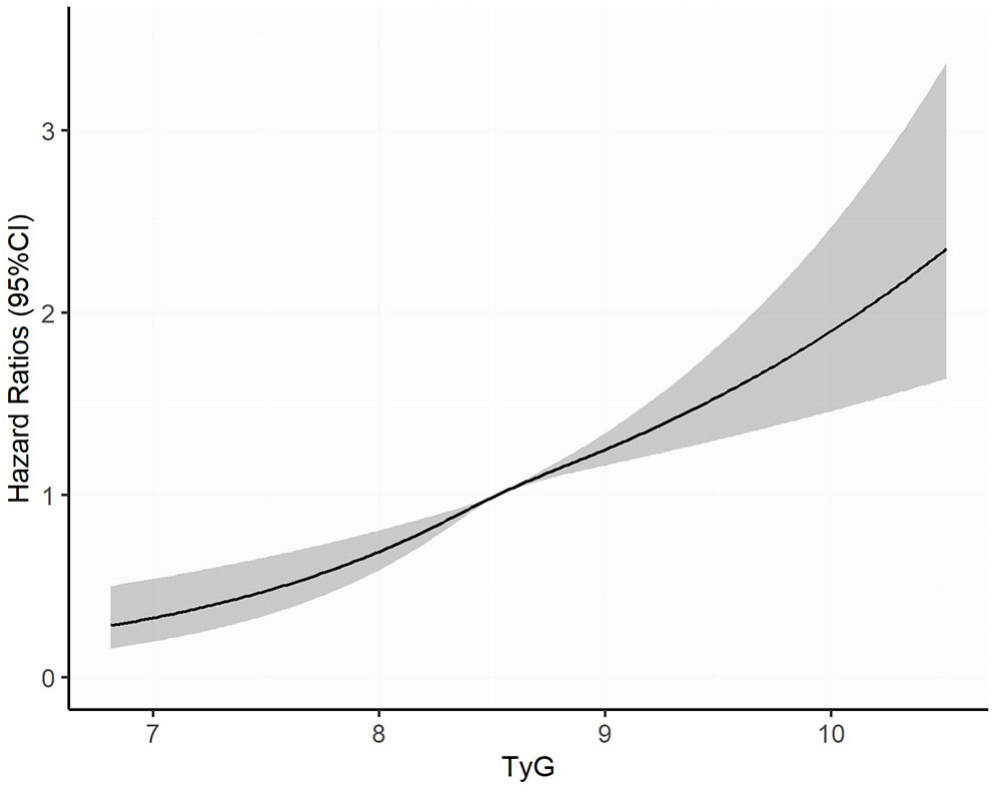
Adjusted cubic spline model of the association between triglyceride glucose index and risk of new-onset diabetes. TyG, triglyceride glucose.

Over a median of 3.4 years of follow-up, 791 (10.6%) participants have developed diabetes. After controlling for age, gender, education, marriage, smoking, drinking, BMI, WC, SBP, history of hypertension, history of CVD, and usage of lipid-lowering drugs (Model 3), every SD increase in TyG index was associated with 22% higher risk of developing diabetes (HR 1.22, 95% CI, 1.14–1.31). When comparing with the lowest quartile of TyG index, the multivariate HRs for new-onset diabetes were 1.22 (0.96–1.54) for Q2, 1.61 (1.28–2.01) for Q3, and 1.73 (1.38–2.16) for Q4 (*P* for trend <0.001) (Table 3). In the restricted cubic spline regression models, the relationship between TyG index and risk of incident diabetes was linear (Figure 2).

**Table 3:** Cox-proportional hazard models for the association between triglyceride glucose index and incident diabetes.

	**Case/total**	**Model 1**	**Model 2**	**Model 3**
TyG index				
Per SD increase		1.34 (1.25, 1.43)	1.35 (1.26, 1.44)	1.22 (1.14, 1.31)
Quartiles				
Q1	123/1,857	Ref	ref	ref
Q2	166/1,857	1.37 (1.08, 1.73)	1.37 (1.08, 1.73)	1.22 (0.96, 1.54)
Q3	232/1,857	1.91 (1.54, 2.38)	1.90 (1.53, 2.37)	1.61 (1.28, 2.01)
Q4	270/1,857	2.27 (1.84, 2.81)	2.32 (1.87, 2.87)	1.73 (1.38, 2.16)
*P* for trend		<0.001	<0.001	<0.001

*Data are presented as hazard ratio (95% confident interval). TyG, triglyceride glucose; SD, standard deviation; Q, quartiles*.

*Model 1 adjust for none*.

*Model 2 adjust for age and gender*.

*Model 3 adjust for age, gender, education, marriage, smoking, drinking, body mass index, waist circumference, systolic blood pressure, history of hypertension, history of cardiovascular disease, and usage of lipid-lowering drugs*.

### Subgroup Analyses

We performed subgroups analyses to stratify the association between TyG index and diabetes by age, gender, BMI, glycemic status, FBG and TG levels, as provided in Table 4. No interaction was found between subgroup variables and association of TyG index with the risk of diabetes (Table 4). The positive relationship remained consistent in both men and women, younger and elderly participants, lean and overweight/obese participants, normoglycemic and prediabetic participants, and participants with or without elevated FBG or TG levels (Table 4).

**Table 4:** Multivariable-adjusted hazard ratios for the association between quartiles of triglyceride glucose index and incident diabetes by subgroups.

	**Case/total**	**Q1**	**Q2**	**Q3**	**Q4**	***P* for trend**	***P* for interaction**
Age, years							0.74
≥65	258/2,029	ref	1.22 (0.81, 1.83)	1.66 (1.13, 2.43)	1.82 (1.23, 2.69)	<0.001	
<65	533/5,399	ref	1.20 (0.89, 1.61)	1.55 (1.17, 2.05)	1.66 (1.26, 2.19)	<0.001	
Gender							0.14
Male	351/3,457	ref	1.15 (0.84, 1.58)	1.33 (0.97, 1.82)	1.56 (1.14, 2.14)	0.004	
Female	440/3,971	ref	1.34 (0.93, 1.94)	1.96 (1.40, 2.75)	1.99 (1.42, 2.79)	0.001	
BMI, kg/m^2^							0.66
≥24	405/2,822	ref	1.53 (1.01, 2.33)	1.85 (1.25, 2.74)	2.03 (1.39, 2.97)	<0.001	
<24	377/4,493	ref	1.08 (0.80, 1.46)	1.55 (1.17, 2.06)	1.63 (1.20, 2.21)	<0.001	
Glycemic status							0.41
Normoglycemia	528/3,888	ref	1.16 (0.82, 1.63)	1.54 (1.12, 2.11)	1.38 (1.01, 1.89)	0.03	
Prediabetes	263/3,540	ref	1.17 (0.84, 1.65)	1.35 (0.95, 1.90)	1.80 (1.25, 2.60)	0.002	
FBG, mg/dL							0.602
≥100	508/3,762	ref	1.16 (0.88, 1.54)	1.26 (0.96, 1.66)	1.40 (1.07, 1.84)	0.013	
<100	283/3,666	ref	1.22 (0.84, 1.78)	1.29 (0.89, 1.87)	1.60 (1.12, 2.29)	0.001	
TG, mg/dL							0.089
≥200	123/825	ref	1.06 (0.61, 1.85)	1.23 (0.71, 2.12)	1.80 (1.09, 2.99)	0.016	
<200	668/6,603	ref	1.23 (0.96, 1.59)	1.44 (1.13, 1.84)	1.74 (1.37, 2.21)	<0.001	

*Data are presented as hazard ratio (95% confident interval). Q, quartiles; BMI, body mass index; FBG, fasting blood glucose; TG, triglyceride*.

*Models are adjusted for age, gender, education, marriage, smoking, drinking, body mass index, waist circumference, systolic blood pressure, history of hypertension, history of cardiovascular disease, and usage of lipid-lowering drugs except the subgroup variable itself*.

## Discussion

From the national data from the CHARLS, we have found a positive relationship between the simple surrogate of IR (TyG index) and the risk of new-onset diabetes in middle-aged and older Chinese adults. The effects of TyG index on diabetes did not interact with age, gender, BMI, glycemic status, FBG, or the level of TG.

Our results were consistent with previous studies that indicated a linear relationship between TyG index and the risk of diabetes. A recent study of 4,285 middle-aged and older Korean adults with BMI <25 kg/m^2^ has found a positive association of TyG index and diabetes after 12 years of follow-up (15). Similarly, in lean Chinese individuals, Zhang et al. suggested that TyG index could predict the risk of incident diabetes (11). The authors claimed that lean people were more likely to suffer from hypertriglyceridemia because of the lack of subcutaneous fat, leading to subsequent IR and β-cell dysfunction (20). In a White European population, Navarro-Gonzalez et al. reported that the risk of developing diabetes was increased by 54% for per SD increase of TyG index, and the authors also suggested that TyG index was a better predictor of diabetes than TG or FBG separately (12).

In the subgroup analyses, we found that the positive relationship between TyG index and diabetes was consistent in all subgroup variables, and seemed to be more evident in elderly, women, obese or prediabetic individuals. The reasons could be explained as follows. Visceral adiposity tissue increases with age and may lead to the higher incidence and risk of diabetes (21). In addition, the higher hepatocellular lipids in women may contribute to the increased risk of diabetes (22). Moreover, a recent study showed that TyG was an important mediator in the BMI-related diabetes development in both obese and non-obese individuals (23). Another retrospective study of 2,900 Korean adults reported that TyG index of 8.8 or higher significantly increased the risk of type 2 diabetes regardless of BMI range (13). Finally, prediabetes is more likely related to IR than normoglycemia, which explains the more pronounced risk of developing diabetes in this population (24–26).

Several mechanisms have been reported to explain the relationship between TyG index and diabetes. On one hand, increased TG level in the blood contributes to the inhibited insulin activity, production of inflammatory cytokines, and muscle catabolism while overloaded TG in the pancreatic islet cells can disrupt the β-cell function (27). On the other hand, elevated glucose concentrations exerts toxic effects on β-cells by raising the level of reactive oxygen species (28). These mechanisms have been confirmed in an intervention study indicating that the capacity of insulin secretion and IR status were improved by the reduction in TG and FBG level (29). As a product of TG and FBG, high TyG index reveals both seriously decreased β-cells and the increased IR, which are considered to be the major stages of diabetes development (30). Despite the proposed pathways, more mechanistic researches are needed to reveal the role of TyG in the development of diabetes in different ethnicities.

Our findings have several clinical implications. First, TyG index was recently reported to be superior to traditional predictors of IR and diabetes such as TG/HDL-C and HMOA-IR (31). Second, several studies have shown that TyG index is a better predictor of diabetes compared with FBG or TG itself, as well as single lipid markers such as TC, LDL-C, and HDL-C cross different ethnic groups (12, 32, 33). Third, TyG index is a simple, inexpensive and routine indicator for clinical practice. Finally, and most importantly, monitoring the TyG index can help to identify people at high risk of developing diabetes, even though their FBG or TG is not high or is at a borderline high level. For this group of people, timely lifestyle and diet adjustments are crucial (34).

### Limitations

The strength of the current research was to include a nationally representative sample, using rigorous and standard protocol for data collection and follow-up. However, some limitations should be considered for cautious interpretation. First, residual confounding might exist such as physical activity and the family history of diabetes. Second, 2-h oral glucose tolerance test was not performed to detect cases of diabetes, which might underestimate the incidence. Third, we could not differentiate statins and fibrates from lipid-lowering drugs from the information in CHARLS. The impact of statins treatment on the association between TyG index and diabetes was not fully addressed, considering that statins treatment might increase the risk of developing diabetes (35). Fourth, the follow-up time was relatively short. Previous studies have suggested that prediabetes was associated with an increased risk of developing diabetes as well as CVD (36, 37), so studies with longer follow-up duration are needed to explore the association between TyG index and risk of CVD. Finally, all participants were Chinese people aged 45 years or older, caution should be made when interpreting our findings in younger individuals and in other ethnic populations.

In conclusion, TyG index was significantly associated with the risk of new-onset diabetes in middle-aged and older adults. TyG index might be a useful marker for predicting new-onset diabetes.

## Data Availability Statement

The datasets presented in this study can be found in online repositories. The names of the repository/ repositories and accession number(s) can be found at: http://charls.pku.edu.cn/en.

## Ethics Statement

The studies involving human participants were reviewed and approved by the Ethics Review Committee of Peking University. The patients/participants provided their written informed consent to participate in this study.

## Author Contributions

C-lC, Y-qH, and Y-qF conceived and designed the study. C-lC, LL, Y-qH, and Y-qF analyzed and interpreted the data. C-lC and Y-qF wrote and/or edited the manuscript. All authors contributed to the article and approved the submitted version.

## Conflict of Interest

The authors declare that the research was conducted in the absence of any commercial or financial relationships that could be construed as a potential conflict of interest.
